# Tuberculosis Test Usage and Medical Expenditures from Outpatient Insurance Claims Data, 2013

**DOI:** 10.1155/2017/3816432

**Published:** 2017-10-23

**Authors:** Kwame Owusu-Edusei, Carla A. Winston, Suzanne M. Marks, Adam J. Langer, Roque Miramontes

**Affiliations:** Division of Tuberculosis Elimination, Centers for Disease Control and Prevention, Atlanta, GA, USA

## Abstract

**Objective:**

To evaluate TB test usage and associated direct medical expenditures from 2013 private insurance claims data in the United States (US).

**Methods:**

We extracted outpatient claims for TB-specific and nonspecific tests from the 2013 MarketScan® commercial database. We estimated average expenditures (adjusted for claim and patient characteristics) using semilog regression analyses and compared them to the Centers for Medicare and Medicaid Services (CMS) national reimbursement limits.

**Results:**

Among the TB-specific tests, 1.4% of the enrollees had at least one claim, of which the tuberculin skin test was most common (86%) and least expensive ($9). The T-SPOT® was the most expensive among the TB-specific tests ($106). Among nonspecific TB tests, the chest radiograph was the most used test (78%), while chest computerized tomography was the most expensive ($251). Adjusted average expenditures for the majority of tests (≈74%) were above CMS limits. We estimated that total United States medical expenditures for the employer-based privately insured population for TB-specific tests were $53.0 million in 2013, of which enrollees paid 17% ($9 million).

**Conclusions:**

We found substantial differences in TB test usage and expenditures. Additionally, employer-based private insurers and enrollees paid more than CMS limits for most TB tests.

## 1. Introduction

In spite of remarkable declines in tuberculosis (TB) incidence in the United States over the last six decades, TB continues to be a public health problem [1]. The major components of TB prevention and control efforts are timely identification of active TB cases, testing for TB infection among high-risk persons, and appropriate treatment of persons identified with TB disease or TB infection. In the United States, the majority (77%) of TB prevention and control efforts (mainly, diagnostic and treatment services) are provided by the public sector through local health departments [2]. However, with the introduction of the Affordable Care Act, provision of health care services to diagnose and manage TB disease through the private sector, financed by private insurance, is likely to increase [2].

The purpose of this study was to examine TB test usage and associated medical expenditures by the private sector using outpatient insurance claims data available from the Truven Health MarketScan Commercial Claims Database. Estimates of private sector usage of individual TB tests will provide information on the relative contribution of the private sector in providing these services. Estimates of the average expenditures on each test are needed for budgeting and cost projections for future testing and diagnosis programs. They are also needed for estimating the overall cost of TB prevention and control activities. Additionally, they are indispensable inputs for conducting economic evaluations—such as cost-effectiveness and cost-benefit analysis—of TB testing and prevention programs. Comparisons of private versus public insurance expenditures on TB tests provide measures of price differentials that can help to determine the economic cost of testing. Finally, results from this study will provide TB test usage and medical expenditure benchmarks that can be useful for assessing trends.

## 2. Materials and Methods

We used retrospective data on private insurance claims for reimbursement (henceforth referred to as “claims”) from the Truven Health MarketScan Commercial Claims Database for 2013. The MarketScan database includes health insurance claims across the continuum of health care (including inpatient, outpatient, and outpatient pharmacy) and enrollment data from large employers and health insurance plans across the United States [3]. The 2013 data contained claims information on more than 43 million people including employees, their spouses, and dependents (Truven Health MarketScan Commercial Claims Database), representing ≈25% of the estimated employer-based privately insured population in the United States [4].

We compiled a list of current procedural terminology (CPT) codes for testing methods used to screen for TB infection (tuberculin skin tests [TSTs] and interferon gamma release assays [IGRAs]) as well as tests for active TB disease diagnoses (chest radiograph, chest computerized tomography, sputum smear and culture, nucleic acid amplification, and drug susceptibility testing). These codes were drawn from various sources, including the American Medical Association (AMA) and Centers for Medicare and Medicaid Services (CMS) websites [5, 6]. We categorized CPT codes as representing TB-specific (the target pathogenic species was* Mycobacterium tuberculosis* only) or nonspecific (the target pathogenic species included—or could potentially include—*Mycobacterium tuberculosis*) tests. The list of CPT codes and their description according to AMA [5] is presented in Table 1. We used the brand names for the respective IGRA testing method CPT codes (i.e., QuantiFERON®-TB Gold In-Tube [QFT-GIT] for 86480 and T-SPOT.TB [T-Spot] for 86481) because they were the only IGRA tests approved by the Food and Drug Administration (FDA) as of 2013 [7].

Using the compiled CPT codes, we extracted claims for tests performed on enrollees in 2013 from the outpatient table. We computed claims rate (number of claims per 100,000 enrollees) as the measure of usage for each CPT code using enrollee data from the enrollment table as the denominator. The associated medical expenditures data for each test were the total payment, which included payments by both enrollee and insurance plan, provided with each claim record [3]. As a result, unless otherwise specified, all the expenditures reported (for each test and overall total) in this study are the total payments from insurance plans and enrollees. Next, following previously published methods [8, 9], we deleted claims with payments ≤$1 and determined the average medical expenditures for each test (CPT code)—referred to as the unadjusted average medical expenditures. Deleting claims with payments ≤$1 was necessary given that some encounters contained ≤$1 total payments for capitated services that might not represent the correct reimbursements [3]. The claim records with ≤$1 represented <6% of the overall total. Given the potential for outliers to influence the estimated unadjusted average medical expenditures, we reestimated the average medical expenditures by using a semilog regression that controlled for claim and patient characteristics—referred to as the adjusted average medical expenditures.

In the semilog regression analyses, the dependent variables were the natural logs of medical expenditures (to reduce the influence of outliers) for each test and the independent variables were age, age-squared variable, quantity of services [number of services performed for a claim], gender [male/female], data supplier [employer/health plan], drug benefit [whether or not enrollee's insurance plan included prescription drug coverage], region of the United States [South/Northeast/North/Central/West/unknown], and health plan type [comprehensive/exclusive provider organization (EPO)/health maintenance organization (HMO)/point of service (POS)/preferred provider organization (PPO)/consumer driven health plan (CDHP)/high deductible health plan (HDHP)] based on previous studies [8–12]. For easy interpretation of the regression results, the coefficients from the regression analyses were transformed as coefficient (*β*)*∗*100 and interpreted as the relative change (in percentage) per unit change in the independent variable (IV) for the continuous IVs and as (*e*^*β*^ − 1)*∗*100 and interpreted as the relative difference (in percentage) in the estimated medical expenditures when compared with the referent category [13].

We examined differences in usage among the TB-specific and nonspecific tests by using 2-sided *z* test for differences in claims rates and used the regression analysis to test for differences in average medical expenditures between tests by including them as categorical variables (equal to 1, 0 otherwise). The adjusted average medical expenditures for each CPT were compared to the CMS fee schedule national limit for 2013 [6] and presented as relative difference in percentages. We also compared the adjusted estimates among the three commonly used TB-specific tests—86480 (QFT-GIT), 86481 (T-Spot), and 86580 (TST). We determined the number of enrollees who had at least one claim for the TB-specific tests and estimated the proportion tested for TB. Finally, we used straight-line extrapolation to estimate the overall total medical expenditures of the TB-specific tests for the entire United States population who had employer-based health insurance in 2013. Because of the potential for overestimation of costs attributable to TB, we did not include the overall total medical expenditures for the nonspecific TB tests. We also estimated the overall total medical expenditures for the three commonly used TB-specific tests—86480 (QFT-GIT), 86481 (T-Spot), and 86580 (TST).

We used MEDSTAT DataProbe version 3.3.15 (Truven Health Analytics Inc., Ann Arbor, MI) for data extraction. Microsoft Excel, version 2013 (Microsoft Corporation, Redmond, WA), was used for summary results and graphic presentations and STATA version 14.0 (StataCorp LP, College Station, TX) was used for all regression analyses including result validation and diagnostics.

## 3. Results

### 3.1. TB-Specific Test Usage

**Figure 1:**
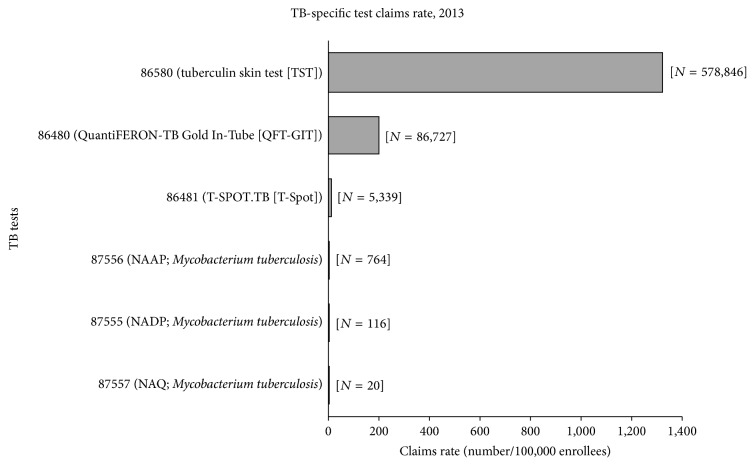
Estimated claims rates (number/100,000 enrollees) for the TB-specific tests. TB, tuberculosis; NAAP, nucleic acid amplification probe; NADP, nucleic acid direct probe; NAQ, nucleic acid quantification. The brand names for the respective IGRA testing method CPT codes (i.e., QuantiFERON-TB Gold In-Tube [QFT-GIT] for 86480 and T-SPOT.TB [T-Spot] for 86481) were used because they were the only IGRA tests approved by the Food and Drug Administration (FDA) as of 2013 [7].

The estimated claims rates for the TB-specific tests are presented in Figure 1. Based on the data from the 2013 outpatient table, the estimated claims rates ranged from 0.05/100,000 (87557 [nucleic acid, quantitative (NAQ);* Mycobacteria tuberculosis*]) to 1,323/100,000 (86580 [TST]) (Figure 1). Overall, TSTs made up 86.2% of the TB-specific tests and similarly 86.3% among the three commonly used TB-specific tests (QFT-GIT, T-Spot, and TST). The claims rate for 87557 (NAQ;* Mycobacteria tuberculosis*) was significantly (*p* < 0.01) lower than the claims rates for all the other TB-specific tests. Conversely, the claims rate for 86580 (TST) was significantly (*p* < 0.01) higher than the claims rates for all the other TB-specific tests (Figure 1). We found that 609,394 enrollees had at least one claim for TB-specific tests, representing 1.4% of the total number of enrollees in 2013.

### 3.2. Nonspecific TB Test Usage

**Figure 2:**
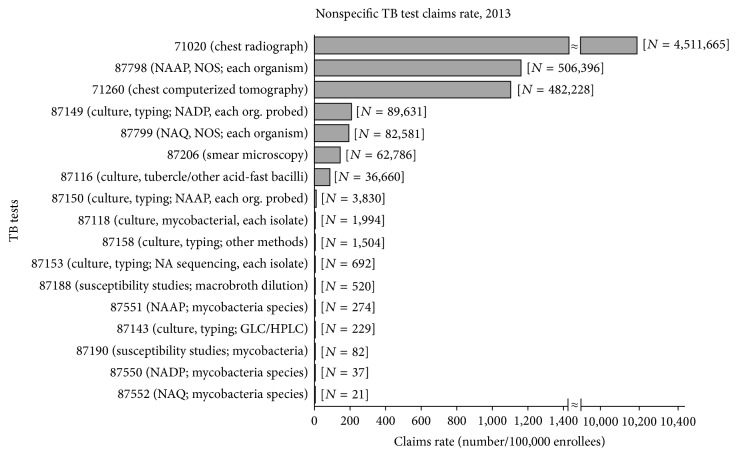
Estimated claims rates (number/100,000 enrollees) for the nonspecific TB tests. TB, tuberculosis; NAAP, nucleic acid amplification probe; NADP, nucleic acid direct probe; NAQ, nucleic acid quantification; NA, nucleic acid; NOS, not otherwise specified; GLC, gas liquid chromatography; HPLC, high pressure liquid chromatography.

The estimated claims rates for the nonspecific TB tests are presented in Figure 2. Among the nonspecific TB tests, the estimated claims rates ranged from 0.05/100,000 (87552 [nucleic acid, quantitative (NAQ); mycobacteria species]) to 10,315/100,000 (71020 [chest radiograph; 2 views]) (Figure 2). Overall, 71020 (chest radiograph; 2 views) made up over 78% of the nonspecific TB tests. The estimated claims rate for upstream tests in the diagnostic algorithm (such as 71020 [chest radiograph; 2 views]) was significantly higher than the claims rates of tests that might be expected to occur later in a diagnostic process (such as 87550 [nucleic acid direct probe (NADP); mycobacteria species] and 87552 [NAQ; mycobacteria species]) (*p* < 0.01) (Figure 2).

### 3.3. Medical Expenditures

Table 2 shows the full regression results used for estimating the adjusted average medical expenditures for the TB-specific tests with the highest usage (86580 [TST]), which included a binary variable to allow for evaluation of the difference in medical expenditures between 86580 (TST) and 86480 (QFT-GIT). Our regression results indicated a quadratic relationship between medical expenditures for the test and age, namely, increased average expenditures by age at a decreasing rate by age (Table 2). In addition, we found that a unit increase in the number of services for a claim resulted in a 16.3% (*p* < 0.01) increase in the associated average medical expenditures for the test. Female enrollees' average medical expenditures were 0.3% (*p* < 0.05) lower than those for males. Average medical expenditures from health plans were 0.6% (*p* < 0.01) lower than those from self-insured employers, and, except for the HMO plan type, all the other health plan types had higher (*p* < 0.01) medical expenditures, on average. Medical expenditures from the South were 12%–32% (*p* < 0.01) lower than those from the other regions of the United States. Finally, the coefficient (*β*) on the 86480 (QFT-GIT) categorical variable implied that the medical expenditures for 86480 (QFT-GIT) were ≈7 times (i.e., (*e*^*β*^ − 1)*∗*100; *p* < 0.01) higher than that for 86580 (TST) on average (Table 2).

Because of the large number of regression analyses conducted to estimate and to compare the medical expenditures for the 23 tests, we are not able to show full results for the other tests—they are available from the lead author. Except for 87552 (nucleic acid, quantitative (NAQ); mycobacteria species), which had a substantially low number of observations (*n* = 21), the qualitative and quantitative results of the coefficients were largely consistent across all the regression results obtained from the other tests.

### 3.4. Medical Expenditures for TB-Specific Tests

The summary results for the estimated adjusted and unadjusted average medical expenditures for the TB-specific tests are presented in the upper panel of Table 3. The estimated adjusted average medical expenditures ranged from $8.68 (86580 [TST]) to $105.81 (86481 [T-Spot]). The average medical expenditures for 86580 (TST) were significantly lower (*p* < 0.01) than the average medical expenditures for all the other TB-specific tests, while the average medical expenditures for 86481 (T-Spot) were significantly higher (*p* < 0.01) than the average medical expenditures of all the other TB-specific tests. When compared to their respective CMS national limits, the adjusted medical expenditures were generally higher—ranging from 0.4% for 86580 (TST) to 66.1% higher for 87555 (nucleic acid direct probe (NADP);* Mycobacterium tuberculosis*). The exception was 86480 (QFT-GIT) which was 18.8% lower than the CMS national limit (Table 3).

Based on the volume of all the TB-specific tests and their associated medical expenditures, we estimated that the overall total medical expenditures for TB-specific tests performed for these enrollees in 2013 were $13.7 million ($13.6 million when focusing on the three commonly used TB-specific tests—QFT-GIT, T-Spot, and TST). Extrapolating to the entire employer-based privately insured population (i.e., 169.0 million [4]) in the United States, we estimated that the total medical expenditures among the employer-based privately insured population for all the TB-specific tests were $53.0 million in 2013 ($52.6 million for the three commonly used TB-specific tests—QFT-GIT and T-Spot [$30.1 million] and TST [$22.5 million]). Enrollees paid approximately 17% ($9 million) of the overall total medical expenditures for the TB-specific tests.

### 3.5. Average Medical Expenditures for Nonspecific TB Tests

The summary results for the estimated adjusted and unadjusted average medical expenditures for the nonspecific TB tests are presented in the lower panel of Table 3. The estimated adjusted average medical expenditures ranged from $9.08 (87158 [culture, typing; other methods]) to $251.11 (71260 [chest computerized tomography]). The adjusted average medical expenditures for 87158 (culture, typing; other methods) were significantly (*p* < 0.01) lower than the medical expenditures for all the other nonspecific TB tests, while the estimated adjusted average medical expenditures for 71260 (chest computerized tomography) were significantly (*p* < 0.01) higher than the average medical expenditures for all the other nonspecific TB tests. Most of the estimated adjusted average medical expenditures for the nonspecific TB tests were higher than the CMS national limits—12 of the 17 nonspecific TB tests (Table 3). The largest difference was the estimated average medical expenditures for 87188 (susceptibility studies; macrobroth dilution), which were 334.7% higher than the CMS national limit. On the other hand, the average medical expenditures of 87149 (culture, typing; NADP, each org. probed) were 32.9% lower than the CMS national limits (Table 3).

## 4. Discussion

The results from our analysis of TB-associated CPT codes from 2013 health insurance outpatient claims data indicated substantial differences in TB test usage and the associated medical expenditures. Among the TB-specific tests, TST was the most used (86%) and least expensive ($9). The most expensive TB-specific test was the T-Spot test (≈$106), which is consistent with a study from the United Kingdom [14], although they found that T-Spot was less expensive per active TB case averted relative to QFT-GIT. Among the nonspecific TB tests, chest radiograph was the most used test (78%), while chest computerized tomography was the most expensive (≈$251).

The TST has been the standard TB infection test in most parts of the world for over a century [15–17]. Although IGRAs were introduced as alternatives to the TST for the diagnosis of TB infection in the United States over a decade ago [18, 19], our results suggest that TST remains the most widely used test performed among the privately insured population in 2013. In fact, the only test that was used more than TST was the chest radiograph, which is a nonspecific TB test because it is used for the diagnosis/evaluation of a myriad of other conditions including—but not limited to—pneumonia, cardiac conditions, bone abnormalities, and chest wall conditions [20]. In addition, chest radiographs are used not only for investigating the presence of active disease among people with clinical signs, but also for ruling out active disease among asymptomatic people with a current or past record of a positive test for TB infection.

We found that the majority of the estimated medical expenditures were higher than their CMS national reimbursement limits. Five out of the six TB-specific tests were 0.4%–66.1% higher, although the adjusted average medical expenditures of the QFT-GIT test were almost one-fifth lower than the CMS limit. Based on the estimated volume and the associated medical expenditures, we estimated that the total medical expenditures of all the TB-specific tests performed among the entire United States employer-based privately insured population in 2013 were $53.0 million, although almost all (99%) of the estimated total medical expenditures were from three tests—QFT-GIT, T-Spot, and TST. Additionally, although the IGRAs were used far less often than the TSTs (13.7% versus 86.3%) among the three commonly used TB-specific tests (QFT-GIT, T-Spot, and TST), they made up the majority of the overall total expenditures (57%).

## 5. Limitations and Strengths

There are some limitations with the use of administrative claims data for this analysis. The database contains information on select employers/health plans based on their willingness to participate. The commercial claims data includes information on persons aged 0–64 years and the proportion of enrollees who were coded as female in 2013 was similar to the United States general population (51% [21]). However, there were a slightly lower proportion of enrollees from the Southern region than the general United States general population (34% versus 37.5% [21]), although approximately 3% of the enrollees' regions were coded as missing/unknown and the proportions for the other regions were similar to the United States general population. Because the data includes information predominantly on large employers [22] in large cities, a relatively higher proportion of the enrollees were from urban areas. As a result, the data were a convenience sample that may not be generalizable to the entire employer-based privately insured population in the United States. Additionally, information on race/ethnicity and foreign-born status is not available. Thus, our extrapolated estimate of the total medical expenditures for all the TB-specific tests among the entire United States employer-based privately insured population in 2013 is a crude estimate and should be interpreted with caution. Also, as with any administrative database, there may be inaccurate information for one or a combination of reasons, including clinician, data unavailability, or data entry errors [23, 24]. As an example, the region variable (geographic location of primary beneficiary's residence) was coded as missing or unknown in some of the claim records (see Table 2), although for a relatively small proportion of the overall total number of claim records we extracted—3%. We did not include CPT codes for magnetic resonance imaging (MRI) which is sometimes used for TB diagnosis or treatment evaluation [25]. Our analyses do not account for the fact that multiple CPTs codes may pertain to the same individual in the course of testing for TB infection or ruling in or out TB disease diagnosis, nor do we account for the cost of clinic visits or patient opportunity costs in obtaining testing.

The current study has several strengths. First, although the enrollees in the database may not be representative of the employer-based privately insured population, it is one of the largest private insurance databases in the United States. With data on over 43 million enrollees in 2013, the database contains information on ≈25% of the estimated United States population with employer-based private insurance in 2013 (169.0 million people [3]). Secondly, the medical expenditures information provided in the database is payments (not charges), so our estimates represent actual dollar amounts paid for the tests performed for these enrollees in 2013 [3, 22]. Finally, this study adds to the literature by providing relevant private sector perspective on TB test usage and the associated medical expenditures in the United States.

## 6. Conclusion

Our study found substantial differences in TB test usage as well as the associated medical expenditures based on the 2013 data. The information on the unique TB test usage and medical expenditures provides the benchmarks necessary for monitoring private sector testing practices and medical expenditures over time, especially with increased access to private health insurance through the Affordable Health Care Act beginning in 2014. After examining trends in the use of IGRAs and TST among privately insured persons in the United States over a 15-year period (2000–2014), a recent study suggested a gradual shift from the use of TST to IGRAs [26]. Future studies can examine the trends in the individual test payments, including the associated shift, if any, in the total expenditures from TST to IGRAs over time.

## Figures and Tables

**Table 1:** Current procedural terminology (CPT) codes and their description for the TB-specific and the nonspecific TB tests.

CPT code	Description (source: American Medical Association online CPT search [5])
*TB-specific tests*	
86480	Tuberculosis test, cell mediated immunity antigen response measurement; gamma interferon (QuantiFERON-TB Gold In-Tube [QFT-GIT])^†^
86481	Tuberculosis test, cell mediated immunity antigen response measurement; enumeration of gamma interferon-producing T-cells in cell suspension (T-SPOT.TB [T-Spot])^†^
86580	Skin test; tuberculosis, intradermal
87555	Infectious agent detection by nucleic acid (DNA or RNA); *Mycobacterium tuberculosis*, direct probe technique
87556	Infectious agent detection by nucleic acid (DNA or RNA); *Mycobacterium tuberculosis*, amplified probe technique
87557	Infectious agent detection by nucleic acid (DNA or RNA); *Mycobacterium tuberculosis*, quantification

*Nonspecific TB tests*	
71020	Radiologic examination, chest, 2 views, frontal and lateral
71260	Computed tomography, thorax; with contrast material(s)
87116	Culture, tubercle, or other acid-fast bacilli (e.g., TB, AFB, and mycobacteria) any source, with isolation and presumptive identification of isolates
87118	Culture, mycobacterial, definitive identification, each isolate
87143	Culture, typing; gas liquid chromatography (GLC) or high pressure liquid chromatography (HPLC) method
87149	Culture, typing; identification by nucleic acid (DNA or RNA) probe, direct probe technique, per culture or isolate, each organism probed
87150	Culture, typing; identification by nucleic acid (DNA or RNA) probe, amplified probe technique, per culture or isolate, each organism probed
87153	Culture, typing; identification by nucleic acid sequencing method, each isolate (e.g., sequencing of the 16S rRNA gene)
87158	Culture, typing; other methods
87188	Susceptibility studies, antimicrobial agent; macrobroth dilution method, each agent
87190	Susceptibility studies, antimicrobial agent; mycobacteria, proportion method, each agent
87206	Smear, primary source with interpretation; fluorescent and/or acid fast stain for bacteria, fungi, parasites, viruses, or cell types
87550	Infectious agent detection by nucleic acid (DNA or RNA); mycobacteria species, direct probe technique
87551	Infectious agent detection by nucleic acid (DNA or RNA); mycobacteria species, amplified probe technique
87552	Infectious agent detection by nucleic acid (DNA or RNA); mycobacteria species, quantification
87798	Infectious agent detection by nucleic acid (DNA or RNA), not otherwise specified; amplified probe technique, each organism
87799	Infectious agent detection by nucleic acid (DNA or RNA), not otherwise specified; quantification, each organism

^†^The brand names for the respective IGRA testing method CPT codes (i.e., QuantiFERON-TB Gold In-Tube [QFT-GIT] for 86480 and T-SPOT.TB [T-Spot] for 86481) were used because they were the only IGRA tests approved by the Food and Drug Administration (FDA) as of 2013 [7].

**Table 2:** Summary regression results. Dependent variable: log of the medical expenditures of test for 86580 (TST) and 86480 (QFT-GIT). *N* = 628,595.

Independent variables	Coefficient (*β*)^†^ [95% CI]	Transformed coefficient (%) [95% CI]^‡^
Age of patient	0.0048 [0.0045 to 0.0051]^*∗∗∗*^	0.48 [0.45 to 0.51]
Age-squared variable	−0.0000558 [−0.0000603 to −0.0000513]^*∗∗∗*^	−0.0056 [−0.0060 to −0.0051]
Quantity of services	0.16263 [0.1577 to 0.1676]^*∗∗∗*^	16.26 [15.77 to 16.76]
Gender		
Male	Referent	Referent
Female	−0.0032 [−0.0059 to −0.0006]^*∗∗*^	−0.32 [−0.59 to −0.06]
Data supplier		
Employer	Referent	Referent
Health plan	−0.0059 [−0.0091 to −0.0026]^*∗∗∗*^	−0.59 [−0.91 to −0.26]
Drug coverage?		
No	Referent	Referent
Yes	−0.005 [−0.0086 to −0.0014]^*∗∗∗*^	−0.5 [−0.86 to −0.14]
Region		
South	Referent	Referent
Northeast	0.1147 [0.1111 to 0.1183]^*∗∗∗*^	12.15 [11.75 to 12.56]
North Central	0.2134 [0.2092 to 0.2176]^*∗∗∗*^	23.79 [23.27 to 24.31]
West	0.2795 [0.2757 to 0.2833]^*∗∗∗*^	32.25 [31.75 to 32.75]
Unknown	0.1743 [0.1653 to 0.1833]^*∗∗∗*^	19.04 [17.97 to 20.12]
Health plan type		
Comprehensive	Referent	Referent
Exclusive provider organization (EPO)	−0.0692 [−0.083 to −0.0553]^*∗∗∗*^	−6.69 [−7.96 to −5.38]
Health maintenance organization (HMO)	−0.007 [−0.0195 to 0.0056]	−0.7 [−1.93 to 0.56]
Point of service (POS)	−0.0478 [−0.0609 to −0.0348]^*∗∗∗*^	−4.67 [−5.91 to −3.42]
Preferred provider organization (PPO)	−0.0306 [−0.0428 to −0.0183]^*∗∗∗*^	−3.01 [−4.19 to −1.81]
POS with capitation	−0.1314 [−0.1502 to −0.1127]^*∗∗∗*^	−12.31 [−13.95 to −10.66]
Consumer driven health plan (CDHP)	−0.0795 [−0.0928 to −0.0662]^*∗∗∗*^	−7.64 [−8.86 to −6.41]
High deductible health plan (HDHP)	−0.0996 [−0.1129 to −0.0863]^*∗∗∗*^	−9.48 [−10.68 to −8.27]
Expenditures (difference)		
86580 (TST)	Referent	Referent
86480 (QFT-GIT)	2.0627 [2.0587 to 2.0666]^*∗∗∗*^	686.72 [683.58 to 689.79]

Significance levels: ^*∗∗∗*^*p* < 0.01; ^*∗∗*^*p* < 0.05. ^†^The *t*-values associated with the coefficients were derived from bootstrap-generated standard errors with 50 replications. ^‡^Because the dependent variables in the regression analyses were the natural log of dependent variable (medical expenditures), the coefficients were transformed as coefficient (*β*)*∗*100 and interpreted as the relative change (in percentage) per unit change in the independent variable (IV) for the continuous IVs and as (*e*^*β*^ − 1)*∗*100 and interpreted as the relative difference (in percentage) in the estimated medical expenditures when compared with the referent (or omitted) category [13]. The brand names for the respective IGRA testing method CPT codes (i.e., QuantiFERON-TB Gold In-Tube [QFT-GIT] for 86480 and T-SPOT.TB [T-Spot] for 86481) were used because they were the only IGRA tests approved by the Food and Drug Administration (FDA) as of 2013 [7].

**Table 3:** Summary results showing the unadjusted and adjusted average medical expenditures for all the tests (2013 United States dollars).

Test	Unadjusted average medical expenditures [SD]	Adjusted average medical expenditures^†^ [95% CI]	CMS limit	Relative difference from CMS
*TB-specific tests*				
86481 (T-SPOT.TB [T-Spot])	140.77 [104.52]	105.81 [103.72–107.94]^*∗∗*^	102.99	2.7%
87556 (NAAP; *Mycobacterium tuberculosis*)	123.34 [141.54]	74.45 [68.53–80.87]	48.24	54.3%
87557 (NAQ; *Mycobacterium tuberculosis*)	77.53 [25.84]	73.1 [68.09–78.47]	58.88	24.1%
86480 (QuantiFERON-TB Gold In-Tube [QFT-GIT])	86.96 [88.08]	69.14 [68.83–69.46]^*∗∗*^	85.20	−18.8%
87555 (NADP; *Mycobacterium tuberculosis*)	73.77 [75.4]	45.78 [37.51–55.88]^*∗∗*^	27.57	66.1%
86580 (tuberculin skin test [TST])	10.52 [26.59]	8.68 [8.67–8.69]	8.64	0.4%

*Nonspecific TB tests*				
71260 (chest computerized tomography)	454.23 [573.78]	251.11 [250.33–251.89]^*∗∗*^	266.40	−5.7%
87153 (culture, typing; NA sequencing, each isolate)	193.31 [149.87]	145.08 [135.66–155.15]	158.57	−8.5%
87150 (culture, typing; NAAP, each org. probed)	275.85 [396.52]	135.04 [130.26–140]^*∗∗*^	48.24	179.9%
87552 (NAQ; mycobacteria species)	127.85 [88.91]	92.66 [52.48–163.61]	58.88	57.4%
87799 (NAQ, NOS; each organism)	122.78 [307.61]	78.86 [78.36–79.36]	58.88	33.9%
87550 (NADP; mycobacteria species)	84.55 [85.42]	60.42 [45.03–81.07]	27.57	119.1%
87798 (NAAP, NOS; each organism)	78.58 [138.91]	47.54 [47.44–47.63]	48.24	−1.4%
87551 (NAAP; mycobacteria species)	84.12 [221.03]	45.89 [41.94–50.21]^*∗*^	48.28	−5.0%
87188 (susceptibility studies; macrobroth dilution)	125.81 [207.41]	39.65 [35.72–44.01]^*∗∗*^	9.12	334.7%
71020 (chest radiograph; 2 views)	63.39 [101.58]	38.16 [38.13–38.2]	30.96	23.3%
87143 (culture, typing; GLC/HPLC)	60.96 [61.75]	37.08 [32.77–41.96]^*∗*^	17.22	115.3%
87116 (culture, tubercle/other acid-fast bacilli)	57.36 [91.95]	31.32 [30.94–31.71]^*∗∗*^	14.85	110.9%
87190 (susceptibility studies; mycobacteria)	51.69 [70.78]	24.8 [18.56–33.13]	7.77	219.1%
87118 (culture, mycobacterial, each isolate)	37.62 [50.52]	20.35 [19.38–21.37]^*∗∗*^	15.04	35.3%
87149 (culture, typing; NADP, each org. probed)	25.35 [75.55]	18.51 [18.44–18.57]^*∗∗*^	27.57	−32.9%
87206 (smear microscopy)	25 [52.12]	12.69 [12.58–12.8]^*∗∗*^	7.39	72.4%
87158 (culture, typing; other methods)	14.53 [28.34]	9.08 [8.7–9.48]	7.19	26.3%

^†^Arranged in decreasing order of magnitude. Significance levels: ^*∗∗*^*p* < 0.01, ^*∗*^*p* < 0.05 higher than the next estimate. CMS, Centers for Medicare and Medicaid Services; SD, standard deviation; TB, tuberculosis; NAAP, nucleic acid amplification probe; NADP, nucleic acid direct probe; NAQ, nucleic acid quantification; NA, nucleic acid; NOS, not otherwise specified; GLC, gas liquid chromatography; HPLC, high pressure liquid chromatography. The brand names for the respective IGRA testing method CPT codes (i.e., QuantiFERON-TB Gold In-Tube [QFT-GIT] for 86480 and T-SPOT.TB [T-Spot] for 86481) were used because they were the only IGRA tests approved by the Food and Drug Administration (FDA) as of 2013 [7].
